# Association of Tumor Lysis Syndrome and Metastatic Melanoma

**DOI:** 10.7759/cureus.18108

**Published:** 2021-09-19

**Authors:** Neil Kelkar, Jue Wang

**Affiliations:** 1 Oncology, University of Arizona College of Medicine - Phoenix, Phoneix, USA; 2 Genitourinary Oncology, Dignity Health Cancer Institute, St. Joseph’s Hospital and Medical Center, Phoenix, USA; 3 Genitourinary Oncology, Creighton University School of Medicine, St. Joseph's Hospital and Medical Center, Phoenix, USA

**Keywords:** tumor-lysis syndrome, malignant melanoma metastasis, tumor lysis in solid tumors, mortality in melanoma, immuno-checkpoint inhibitor

## Abstract

Background

Tumor lysis syndrome (TLS) is a known oncologic emergency characterized by severe metabolic derangements. TLS has been well documented in patients with hematologic malignancies, but rarely with metastatic melanoma. The objective of this study was to investigate the clinical characteristics and outcomes of TLS with metastatic melanoma.

Methods

Retrospective literature review and analysis.

Results

Eighteen cases of TLS were identified with metastatic melanoma from published literature. The median age of patients was 63 years (36-79 years). All patients have stage IV disease. Seven cases (39%) of TLS were associated with multiple treatment regimes, including nivolumab (22%), ipilimumab (16%), and dacarbazine (22%). The time from treatment to diagnosis was 3.5 days (8 hours-21 days) in treatment-related TLS. Three cases (17%) were due to spontaneous TLS. The majority of cases have a high tumor burden (77.5%) and liver metastasis (83%). Seven cases were treated with rasburicase (39%). The mortality rate was 100% for the patients with spontaneous TLS and 73% for patients with treatment-related TLS. Three cases utilized traditional chemotherapy and the six most recent cases of treatment-associated TLS utilized immunotherapy and targeted therapy.

Conclusion

TLS in metastatic melanoma, due to either spontaneous or treatment-related causes, is associated with a very high mortality rate. This study highlights the importance of awareness, early intervention, and risk assessment of this underdiagnosed emergency.

## Introduction

Tumor lysis syndrome (TLS) is a serious, life-threatening oncological emergency due to the massive cellular lysis and release of contents into the bloodstream. This results in severe metabolic derangement including hyperkalemia, hyperphosphatemia, hyperuricemia, and hypocalcemia [1]. Although TLS is a well-characterized complication for hematologic malignancies such as acute lymphoblastic leukemia, it is underreported in solid tumors [2]. Between 1994 and 2020, we identified 18 different cases of TLS associated with malignant melanoma [3-18]. The objective of this study was to examine published information on management, characteristics, and outcomes of patients with TLS associated with malignant melanoma.

## Materials and methods

Literature search

This is a retrospective systematic review of published cases of patients with metastatic cancer who were diagnosed with TLS. This was performed by searching the database PubMed for articles published from January 1991 to May 2021. Search terms included “tumor lysis syndrome,” “solid tumor,” and “melanoma.” The resulting case reports were reviewed, and additional articles of interest were identified from reference lists.

Data collection and analysis

Information regarding the patients including gender, age at diagnosis, presentation, associated medical history (such as prior renal function), tumor characteristics (symptoms, pathology, stage), treatment, laboratory results, and outcomes (response, adverse effects) were recorded when available. Statistics such as medians, ranges, and frequency counts were used to assess the pooled sample.

## Results

A total of 18 cases of metastatic melanoma-related TLS (15 cases treatment-related and three cases of spontaneous TLS [sTLS]) were identified from 17 case reports. The demographic, symptoms, and survival outcomes are summarized in Table 1. 

**Table 1:** Overview of published cases of tumor lysis syndrome in patients with metastatic melanoma

Author	Year	Age/Gender	Treatment	Liver Metastasis	Rasburicase	Outcome
Minasian [3]	1994	76/M	Tumor necrosis factor-a, anti-GD3 ganglioside MoAb	Yes	No	Death
Castro [4]	1999	61/M	IL-2, interferon-a, cisplatin, vinblastine, and dacarbazine	Yes	No	Death
Stoves [5]	2001	41/M	Cisplatin, interferon-a, dacarbazine	Yes	No	Death
Habib [6]	2002	56/F	Hydrocortisone	No	No	Survival
Busam [7]	2004	36/F	Cisplatin, vinblastine, dacarbazine, interferon alpha	Yes	No	Survival
Borne [9]	2009	42/M	High-dose steroids	Yes	Yes	Death
Song [10]	2011	46/M	Spontaneous	Yes	No	Death
Mouallem [11]	2013	69/M	Spontaneous	Yes	No	Death
Mouallem [11]	2013	68/M	Prednisone and dacarbazine	Yes	No	Death
Dar [12]	2014	65/M	Palliative radiation therapy	Yes	Yes	Death
Meeks [18]	2016	46/M	Dexamethasone	Yes	Yes	Death
Masson [8]	2017	71/M	Ipilimumab	Yes	Not recorded	Death
Brunnhoelzl [14]	2018	76/M	Nivolumab	Yes	Yes	Death
Valle [15]	2019	65/M	Spontaneous	Yes	Yes	Death
Sugimoto [16]	2020	79/M	Nivolumab	Yes	No	Death
Magara [17]	2020	45/M	Nivolumab + ipilimumab	No	Yes	Survival
Byron [13]	2020	48/F	Encorafenib + binimetinib	No	Yes	Survival
Konishi [19]	2020	69/M	Nivolumab + ipilimumab	Yes	Yes	Death

Patients in the study had a median age of 63 years (36-79) and showed a male predominance (77.6%). Majority of patients presented with liver metastasis (83%) and high tumor burden (77.5%). Patients were noted to have elevated lactate dehydrogenase (LDH) with other biochemical indications of TLS such as uric acid, potassium, and phosphorus. Fifteen cases (83%) of TLS were associated with a variety of treatment options including nivolumab (22%), ipilimumab (11%), dacarbazine (22%), cisplatin (17%), vinblastine (11%), and interferon-a (11%).

The median time from treatment to diagnosis of TLS was 3.5 days (8 hours-21 days). Three cases (17%) were due to sTLS. Seven cases were treated with rasburicase (39%). The mortality rate was 73% for patients with treatment-related TLS and 100% for patients with sTLS.

## Discussion

TLS is a well-known emergency in hematological malignancies. TLS was considered rare in solid tumors, as effective pharmacological treatments were not available. However, rates have been significantly increasing as therapies become more effective [2]. TLS can hypothetically occur in every tumor type, as the tumor burden is considered a more important factor than the origin of the tumor tissue (18). High tumor burden is defined as having three specific measurements: elevated LDH (>2x the upper limit of normal), bulky disease (>10 cm), and elevated leukocytes (>25,000). Studies have also characterized tumor burden by the evidence of distant metastases [20,21]. Our study found that all studies, which reported an LDH reading, had a reading above two times the upper normal limit.

Melanoma is the least common, but the most malignant lesion of the skin. The mortality rate of melanoma differs with age and gender, but recent studies place the rate around 2.7/100,000 cases [22]. However, TLS due to melanoma is frequently misdiagnosed. TLS is often misclassified as acute kidney injury or other causes of electrolyte abnormalities. Additionally, adverse effects of pharmaceuticals during clinical trials are frequently underreported [23]. We believe that the incidence of TLS due to melanoma is underreported and underdiagnosed, and, therefore, prevents clinicians from understanding the true epidemiology of this emergency [3-17].

The study outlines several notable findings. First, TLS in metastatic melanoma carries a worse prognosis when compared to hematological malignancies. TLS morality for hematological cancer is approximately 21% for hematological cancers, but our study shows a mortality rate of 73% for treatment-induced TLS and 100% for sTLS [24]. This is likely due to a lack of awareness of TLS in metastatic melanoma, and other solid tumors. The result is a delayed diagnosis and inadequate management, leading to a higher mortality rate for this population. Second, TLS commonly occurs in patients who have advanced cancer with large disease burdens. Liver metastases were documented in 17 patients (Table 1), showing an increased risk of developing TLS [25]. The most important finding from examining studies is that TLS due to metastatic melanoma occurs at a median of 3.5 days with a range of 8 hours to 21 days. This suggests that TLS rapidly develops due to effective therapies. For patients who are at increased risk of developing TLS, we advise monitoring patients’ electrolytes and renal function during the first few weeks of therapy.

Current guidelines and management of TLS, which are based on pediatric and adult hematological malignancies, were established 13 years ago, and at that time, effective therapies for metastatic melanoma and other solid tumors were not available. Our study found that most TLS associated with metastatic melanoma were detected recently when effective treatment was first available. Only three studies utilized traditional chemotherapy. However, 16/18 (89%) cases were reported after the year 2000, and the six most recent cases of treatment-associated TLS utilize immunotherapy and targeted therapy. This suggests that improved treatment modalities have increased rates in solid organ tumors. The mechanism of checkpoint inhibitors, such as ipilimumab, has not fully been revealed. It is believed that checkpoint inhibitors lead to T-cell activation and cause cytokine-dependent endothelial toxicity and massive destruction of tumor cells [14,26].

## Conclusions

This review highlights the nature of TLS, an under-recognized emergency due to metastatic melanoma. It is essential that clinicians recognize that TLS may occur shortly after treatment and spontaneously. There is a need to redefine the risk factors and guidelines of TLS in the era of targeted and immunotherapy.
